# Gleanings from Our Exchanges

**Published:** 1872-11-15

**Authors:** 


### Gleanings from Our Exchanges.

### ERGOTINE AS A HÆMOSTATIC.

BY C. H. BOARDMAN, M.D.

An article in a recent number of *Ranking's Abstract* upon the use of Ergot as a hæmostatic, having attracted my attention, I determined to avail myself of the first suitable opportunity for testing its virtues.

The case reported to which reference has been made, was one of severe pulmonary hæmorrhage. The blood flow was almost instantaneously checked, and its subsequent return prevented by the hypodermic administration of Ergotine, after the failure of other more commonly used remedies.

I was called about the middle of last April to see a lady for whom my services in her approaching confinement had been some time previously engaged, and who was, at this date, at about the middle of the eighth month of term.

I was informed by my patient—who is a woman of fine physique, aged about 28, robust, and capable of great endurance, and who had given birth to three children, her previous labors presenting only the features of ordinary accouchments—that her health and general condition had been, up to this time, excellent, and that she had not been compelled to desist, even for an hour, from her usual domestic duties, which were somewhat onerous. A few weeks previously, however, she had noticed a very slight discharge of blood from the vagina, which would not have been remembered but that it had been followed shortly by another, until now she lost blood nearly every day, and that some of these manifestations were quite copious; sufficiently so to cause a little anxiety on her part, and even, she thought, to induce sensations of weakness or of faintness to a slight degree. They were very apt to follow any effort, however moderate, and often followed any persistence in maintaining an erect position. Hitherto the bleeding had readily yielded to rest in a recumbent posture, or to cold drinks, or to both; but now these means were no longer efficacious. When I first saw my patient at this time, she was lying down, pale, and quite faint; a hæmorrhage of about a half pint having ceased just before. An examination established the fact that a placenta prævia was to be dealt with, and stringent orders were enjoined, such as were appropriate to that condition. The administration of *Ol. Erigeron Philadelphicum* proved efficient for several days in checking the flow as soon as it showed a disposition to recur; and opium occasionally as a suppository both relieved the pelvic pains, now commencing, and also seemed to aid in suppressing the discharge. The beginning of the ninth month was reached thus in safety, and the bleeding seemed so fully under control, that I began to entertain hopes of my patient's accomplishing the full time. But she had found her enforced quietude very irksome, and believing that the hæmorrhage was now completely arrested, she yielded to her urgent desire to enter again upon some at least of her household duties.

Flowing commenced at once, and to a serious extent; former remedies were no longer useful, and I resorted to ergotine. Having no means for measuring it at hand, I poured about two drops into water, enough to render it sufficiently fluid for my purpose. Half of this was injected over the biceps, and in a few minutes, not more than five, the patient spoke of a giddy, throbbing sensation in her head, and the flow of blood ceased abruptly, and as completely as though a tampon had been used. From fifteen to eighteen hours later its threatened return was again as promptly checked; and for some ten days it was completely held in abeyance by this remedy occasionally administered, aided, of course, by absolute quiescence, suitable diet and other means appropriate as adjuvants. Thus for a period of nearly two weeks, the perils incident to this very grave condition were averted, and the patient brought safely to within a fortnight of her full time, where all other resources had failed, and where, but for the ergotine, she must have encountered greater dangers by so much as the enforced confinement might have anticipated the natural period for that event.

It may be said, in conclusion, that a week or ten days before uncomplicated labor would have occurred, hæmorrhage set in, so violent as to defy all efforts for its suppression; the os dilating, had torn away from quite a large segment of the placenta on the left side. The head presenting in the first position, after separating the placenta to a sufficient extent, the forceps were applied, my friend Dr. W. assisting, and the child quickly delivered. It was a male, very large and well developed, but completely asphyxiated by a double coil of the cord about its neck, so as to resist long and vigorous efforts for its resuscitation.

The mother, though reduced and exhausted by loss of blood, especially during labor, to such an extent that fears were entertained as to her recovery, reacted shortly, and possessing remarkable recuperative powers, was again engaged in her household duties in two weeks, and showed no trace of the dangers through which she had so recently passed.—*N. W. Med. and Surg. Jour.*, Nov. 1872.

St. Paul, Oct. 5, 1872.

---

### ELECTRICITY IN THE TREATMENT OF MALIGNANT TUMORS.

Dr. J. S. C. Greene, in the Boston *Medical and Surgical Journal*, writes in reference to late statistics on this point :

Among the sarcomata reported are the two first tumors treated by electrolysis. Each was larger than an apple, and each was destroyed in fifteen minutes, at a single sitting. A sarcoma epulis as large as a pigeon's egg, was, in seven minutes, destroyed down to the bone. No fever, and but slight local reaction followed. On the thirteenth day, the tumor, with a small lamella of bone came away, and cicatrization was rapidly completed.

Of the cases of cancer, the majority were very extensive, some, indeed, quite hopeless. Of the whole number, however, thirteen were successfully operated on, two were discharged from hospital improved, and two without any improvement. Of these, one was a patient who refused to submit to a completion of the operation, because of the pain, anæsthesia having been but incompletely produced (no uncommon occurrence in Vienna opperating theaters, a tacit confession of the distrust in which chloroform is regarded), and one died of incurrent disease during time of treatment. In the cases of the most extensive disease a very powerful current was employed, under anæsthetics, usually in a single sitting, with one re-application to suspicious spots. In other cases, anæsthetics were not employed; the current was used as strong as the patient could bear it, and the operation was protracted to four or six sittings of from fifteen to sixty minutes.

In a case of cancer of the rectum, involving the vicinity of the orifice externally, and extending upwards from the annus two inches, and sharply defined above, the patient suffered from pains so severe that even large doses of morphine injected subcutaneously could only temporarily palliate it. At the same time a most penetrating odor was disseminated. In the first sitting, the external part alone was attacked, and in forty-two minutes it was destroyed. No anæsthetic was used. The pain from this time became so much less, that the patient required no more opiates, and the offensive odor ceased to give further annoyance. The sloughs separated on the fifth day. Three days later the internal infiltration was submitted to treatment. The sloughs separated soon, and the general health of the patient improved so much, that in less than seven weeks from the date of the second operation, he was discharged, well. The subsequent history of this case is not given.

In three cases it was possible thoroughly to destroy carcinoma which was so intimately connected with the inferior maxilla, that, had it been operated on by the knife, resection of the bone would have been necessary. In another case of epithelial cancer of the lower lip, the swelling of the submaxillary glands of both sides, which were largely infiltrated, entirely disappeared after the destruction of the primary disease by electrolysis. The same phenomenon was observed and reported by Dr. Neftel, of New York, in a case of recurrent cancer of the breast, which had been previously twice removed by excision.

It is worthy of note that neoplasmata have been frequently observed to grow much more rapidly under the stimulus of too weak a current, which had been employed with the intention of destroying them.

These may serve as illustrations of some of the advantages of electrolysis as a means of operation. Besides, it should be observed that there is complete absence of loss of blood, and of all reaction due to such loss. Indeed, as a means of arresting bleeding from an artery which is inaccessible to ligatures, nothing can be neater than to pierce the vessel with a needle, just above the bleeding point, the needle being connected with the positive pole of a battery, the negative pole, being, of course, also brought into connection with the body near by.

So free from risk is the application of electrolysis, that Benedict reports a case in which he introduced a long needle into a fibroid tumor of the uterus through the abdominal walls: no general reaction followed, and the local reaction was quite insignificant. So completely free from all formidable features is it, if performed under ether, that electrorysis may be employed, even if only as a paliative operation, in many cases, where it can at least afford relief from painful and distressing symptoms, and render a prolonged life supportable, when operation by the knife might be refused by the patient, or, indeed, might involve so many chances of an unfavorable result as to forbid the surgeon's advising it.

On the other hand, the expense of a battery fitted for electrloytic operations is not inconsiderable; such a battery is with difficulty transported; and with the batteries hitherto used the time which such operations require is relatively somewhat long. Perhaps still more powerful batteries, or the simultaneous use of more than one for different parts of a tumor, might obviate this last disadvantage.—*Phil. Med. Report.*

### TREATMENT OF SO-CALLED URÆMIC CONVULSIONS.

Although we are, in general, no advocates of the wet-packing system of therapeutics, we have been struck by the propriety of its application as stated below, and feel inclined to adopt it.

In the *Philadelphia Medical Times* for Sept. 2, 1872, is a translation of an article by Jaquet, of Berlin, in which he speaks of the skin as the most available medium by which the system may be freed of the convulsion-producing agent, whatever it may be; thus, accepting the uræmic theory, with or without Frerichs' supposed carbonate of ammonia metamorphosis, the skin is by far the best vicarious emunctory when the kidneys have been blocked up; or, accepting the theory of œdema of the brain, by no other route can we so promptly call the pressure of the blood away from the interior of the body, and get rid of its watery elements. Venesection scarcely reduces the bulk of the blood more rapidly, while it possesses the disadvantage of leaving the patient in a more hydræmic condition than it found her.

On these points there is scarcely room for a difference of opinion. The important question is, how can we best produce the requisite diaphoresis? Jaquet resorts to the wet-packing, as employed by Preissnitz, in the following manner:

He takes a woolen blanket, which should be at least seven feet long by six wide, so that the entire body can be completely enveloped. Spreading this out upon an ordinary bed, a sheet of the same size is laid upon it, the sheet having previously been wrung out of cold water, 72 deg. F. The patient is laid upon this and wrapped in the sheet, and then firmly enveloped in the blanket. The head remains free, and is enveloped with a bladder of ice or a cold compress. The chest must not be constricted, but it will be well to keep the legs close together. If labor-pains have already begun, and especially if dilitation of the os uteri be well advanced, it will be necessary to wrap the legs separately, so that we need not unwrap the body for the delivery, which latter, if it should cause a sudden suppression of the perspiration, would readily renew or increase the convulsions. The effects of this wrapping are as follows: during the first two to five minutes, the blood recedes from the periphery to the interior of the body, the skin appearing pale. Ten minutes afterwards a delightful warmth sets in, and the skin having reacted from the impression of the temporary cold, becomes intensely red. After an hour the secretion of sweat begins, without being preceded by any unpleasant feeling of heat or congestion of the head, such as we commonly observe in perspiring in a dry medium. The absence of this phenomenon during the employment of wet wrappings is explained by the fact that water is a better conductor of heat than air, and therefore prevents an unpleasant accumulation of heat in the body. The perspiration now lasts as long as the patient remains wrapped up, being most profuse during the first three or four hours, and then gradually diminishing. The convulsive seizures diminish in number and intensity very soon after the breaking out of the sweat. The loud snoring between the paroxysms ceases, and the patient falls into a quiet sleep. The activity of the uterine contractions continues undisturbed, and hence we can allow the patient to retain her wrappings until delivery is accomplished. Of course the use of wrappings does not interfere with the employment of other remedies of known power, such as chloroform by inhalation.

On account of the restlessness of the parturient woman, and the impossibility of keeping her covered, at the moment of delivery, at least, the method of producing profuse diaphoresis by wet-packing seems to be the only efficient one at our command. In uræmic convulsions from other causes, there are other means perhaps equally efficient. Thus, in the present number of the *Journal*, a case is reported by Dr. Stille where convulsions of this class came on, apparently the result of acute œdema of the brain rather than of retained urinary elements, and in which the happiest results were produced by the conjoined use of chloroform and the hot air bath, both the convulsions and the coma rapidly disappearing as soon as the perspiration became free. The hot-air bath spoken of is applied by raising the coverings slightly from the patient, and tucking them well in around sides of bed, so as to form a sort of tent; a large alcohol lamp is then burned under the flange of a large tin funnel, the nozzle of which is long, bent at a right angle, and opens within the tent of bedclothes. The amount of vapor of water generated by chemical combination in the burning of the alcohol is sufficient to render the hot air moist and pleasant, and thus adds to its diaphoretic power. When well applied, there is almost no limit to the sweat which can be made to pour from the body by this method.

A short time ago we heard of a popular remedy, new to us, but perhaps familiar to our readers. It was called a "corn-sweat," and consisted in immersing about a bushel of ears of corn in boiling water; when well soaked they were removed, wrapped in cloths and packed all around the patient. The amount of heat and moisture that the ears are capable of containing is sufficient to keep up quite a prolonged effect, and seems to answer the indication almost, or quite, as well as the more elegant methods by the wet blanket or the lamp and hot-air pipe.—*N. W. Med. and Surg. Journal.*

Drs. Beard & Rockwell, in a report on the experimental use of electricity in the *Amer. Practitioner*, remark as follows in regard to its use in the removal of tumors:

Of tumors, ten cases were treated, as follows: two cases of scirrhus in the breast, one of epithelioma of the lip, two of uterine fibroid, four of goitre, and one of cystic tumor. The goitres were all reduced in size more or less by the use of the needles; but none have yet been entirely cured, although some of them have been treated with great perseverance and by all varieties of applications, by catalyzation — that is, by external applications with sponges, by one needle, or by many needles — with and without ether, and by ether spray. Goitres do not rapidly disappear under electrolyzation. In many cases, like fibroids and other tumors, they grow smaller under the treatment up to a certain point, when they hang fire, and will not budge an inch further.

It is, of course, possible to destroy any tumor that is accessible by electrolysis, provided that a sufficiently strong current be used, and sufficient time be given to it; but then the question must always arise whether the disease, or the remedy is most to be dreaded. For malignant growths the choice is easy. For benign, painless growths, like small goitres, it is a question whether they are worth the pain and annoyance of an operation of any kind.

*The method of working up the base.*—The epithelioma of the lower lip was destroyed, root and branch, by a method of electrolyzation that we have recently employed, and which we call, *working up the base.* This method consists in inserting the needles around the tumor, and partly into the healthy tissue, so as to undermine the former, and cut off all communication between it and the healthy tissue. This method is followed by complete sloughing of the growth, granulations, and healing. The usual and accepted method of electrolyzation, is to insert the needles directly into the tumor.

This method of working up the base, or undermining the tumor, has the advantage that it is more thorough, since it makes sure of the complete destruction of the growth; that it is shorter, since it wastes no time on the body of the tumor, which really is of no consequence if it be separated from the healthy tissues; and that it insures a more satisfactory healing.

On the Early Diagnosis of Typhoid Fever.—Dr. P.W. Latham, Cambridge (*Atlanta Med. and Surg. Jour.* from *London Lancet*, June, 1872) in a clinical lecture on the subject, remarks as follows:

"From the information the thermometer gives me, I fully endorse the following statement: 'The physician who judges of fever cases without taking note of the temperature, is like a blind man trying to find his way. With much practice and great intelligence, the blind man may succeed; but he will more frequently fail, and always do, only with great difficulty and unsatisfactorily, what to him who sees, requires no effort.

"Let me show you how far this is true. During the first four or five days, the general symptoms which may then, as I told you, accompany the disease—viz., the rigor, the languor and feebleness, headache, epistaxis, giddiness, pain in the back and aching of the limbs, the appearance of the tongue, the state of the bowels, the condition of the urine, etc.—may not be very distinct, or any of these morbid symptoms may be entirely absent. In a considerable number of cases, in fact, it would be impossible for you to say, without using the thermometer, whether the patient were suffering from typhoid fever or not. But the thermometric course of the disease at this time, unless it supervenes on some other malady, is very regular; and by taking the temperature at eight A. M., and at six P. M., for three days, the presence of typhoid fever may be decided. On the other hand, one single observation may, with very great probability, negative the existence of the disease.

"The following is the formula (from Wunderlich) of this initial stage:

| | MORNING. | EVENING. |
|---|---|---|
| First day, | 98.6° F. | 100.4° F. |
| Second day, | 99.4° F. | 101.4° F. |
| Third day, | 100.4° F. | 102.6° F. |
| Fourth day, | 101.6° F. | 104° F. |

"If, then, a person, previously quite well, feels uneasy, perhaps has a rigor, and in the evening we find his temperature about 100.4° or 101° F., falling the next morning about a degree, rising again in the evening, and approximately following the above course, the disease may be diagnosed with tolerable certainty."—*N. W. Med. and Surg. Jour.*

Dry Gangrene following the Use of Carbolic Acid.—M. Poncet, an *interne* of the Lyons Hospital, records in the *Bulletin de Theraputique* (July 30th), a case which he considers rightly to convey a caution against the rash use of carbolic acid by non-professional persons. A girl, aged 13, had her fore-finger injured by a splinter of wood, which penetrated under the nail. The end of the finger was dipped in a bottle of solution of carbolic acid, and a compress soaked in the acid was applied. The next day the part had a greyish color, and was insensible; and when M. Olliver first saw her, a week afterwards, mortification had extended as far as the upper third of the second phalanx, and a line of demarkation was being formed. The finger assumed a mummified appearance, and separated on the thirty-fifth day. This occurrence suggested to M. Ollier the possibility of amputating fingers and toes by means of the application of carbolic acid; and experiments were accordingly made by M. Viennois on rabbits and fowls. The results showed that the application of carbolic acid was liable to be followed by toxic symptoms; but that these might be prevented by the application of a ligature. M. Olliver endeavored to put the plan into execution in a case of disease of the great toe, by plunging it for some minutes in a concentrated solution of carbolic acid; the thickness of the epidermis, however, prevented mortification from being produced. M. Poncet records also the case of a man aged 23, who, having wounded the end of one of his fingers, applied for ten days charpie impregnated with carbolic acid, and afterwards poultices. When he applied to M. Olliver, two months after the accident, the terminal phalanx was in a state of dry gangrene, and was removed a few days afterwards.—*Med. Reporter.*

Dry Earth in Surgery.—Dr. P. S. Conner, of Cincinnati, has been trying the earth treatment, as recommended by Dr. Addinell Hewson, with whom he agrees as to its beneficial effects in the following particulars:

1st. "The constant, universal testimony of patients submitted to the earth dressing, enables me to confirm Dr. H.'s statement, that 'the immediate, or primary, effect of the direct contact of the earth with the part at least cannot be considered irritating.'" "Cool and pleasant," is the constant answer of patients when asked how the application feels.

2d. The patients complain less of the pain naturally incident to the injury, than they do under the use of other dressings.

3d. As a deodorizer it cannot be too highly praised. In midsummer, the surgical wards of the Good Samaritan Hospital have been entirely freed from noxious odor by a careful attention to the proper application of the earth.

4th. That during the application of the earth dressing there is a marked absence of inflammatory redness around the wound. The granulations are very healthy, and free from any tendency to become large and flabby. Suppuration is certainly less than under the bran or water dressing; thus, it has been seen to diminish more than one-half in the first twenty-four hours after the substitution of the dry earth.

He uses yellow, clayey earth, dried and sifted, which should be made to cover the wound to a depth varying from one-fourth to one, or one and a half inches.—*The Clinic.*

Surgical Affections of Adolescents.—M. Gasselin, of Paris, offers the following formula for guidance in the selection of means of treatment in this class of persons. "The special, spontaneous surgical affections of young persons have a tendency to persist, increase or relapse, as long as the period of adolescence lasts ; but these tendencies are lost as soon as adult age is reached." Thus, he says, ingrowing toe-nail will constantly recur up to the age of 23 or 24, but very rarely will do so after the 25th year. Ostitis, exostoses, and fibrous nasopharyngeal polypi, are cited as other examples of the same rule.—*N. W. Med. and Surg. Jour.*

A Substitute for Quinine. — The last number of the *Gazette Med. de Paris* (Sept. 21, '72), details a number of cases of intermittent fever treated by the carbazotate of ammonia. The results were as successful and as speedy as with the administration of quinia. The remedy is given in pills in the dose of two ctgr. a day.

Death from Ether (*Medical Record*, Oct. 1, 1872; by W. B. Dunning, M. D., Acting House Surgeon, Bellevue Hospital).—John Stockander, a German saddler, unmarried, sixty-eight years old, was admitted to Ward thirteen of Bellevue Hospital, on August 2, 1872, suffering from a fracture of left femur, just below the trochanter. The patient was treated by a Buck's Extension until August 20, when it was decided to apply a plaster of Paris splint. In order to make sufficient extension, and at the same time prevent the pain of the operation, ether was ordered to be administered. The administration of the anæsthetic was slowly and carefully made, and after perhaps ten minutes the patient was fully under its influence and the operation begun. A few turns of the plaster had been made, when the patient's breathing was observed to be rather frequent and gasping. The pulse was, however, full and regular. The thorax was compressed two or three times, and the patient's breathing again became normal. As these symptoms not rarely occur during etherization, they excited no special alarm. The ether was, however, withheld from the patient four or five minutes, his respiration and pulse being normal. As he then began, however, to move about, and his muscles were becoming rigid, the ether cone was again applied. In a minute or two, my assistant, who was giving the ether, observed the pupils to be dilating rapidly, and the breathing to cease. His heart was still beating, however. The ether cone was of course immediately removed, and artificial respiration was again used, and all the batteries obtainable in the hospital were put in operation in an effort to resuscitate the patient. His muscles occasionally responded by a spasmodic movement, but no breathing again occurred. The efforts at resuscitation were continued about forty minutes, until all response to the action of the battery had ceased.

Patient died about four P.M., and the autopsy was made at seven P.M. that same day, under direction of Dr. Delafield. Rigor mortis was marked. Blood was fluid. Brain and membranes neither anæmic nor congested. Trachea and larynx somewhat pale. Heart contained a little fluid blood, with a little atheroma at base of aortic valves. Lungs have old adhesions over both. Emphysema exists, and thickening of large bronchi. The lower lobe of right lung is œdematous, and its lower portion in a state of red hepatization. Rest of lung is normal and not congested. Liver is small and firm, containing a good deal of fluid blood. The other organs are normal.

The ether used was that made by Powers & Weightman. It has been examined by Dr. Squibb, of Brooklyn. He states that he "finds nothing in the character or quality of the ether to account for the death of the man, or even to aid in accounting for it." He adds, that in his "judgment the death of this patient is in no way attributable to either the quality of the ether, the quantity used, or the mode of administration, but that it is one of those accidents which, though of very rare occurrence under the careful use of ether, is inseparable from the condition of anæsthesia."

The quantity of ether used was about ℥ vj.

Galvanic Treatment of Bed-Sores and Indolent Ulcers.—Dr. Wm. A. Hammond recommends for indolent ulcers and bed-sores the galvanic treatment as first recommended by Crussel, of St. Petersburg. He says: "During the last six years I have employed it to a great extent in the treatment of bed-sores caused by diseases of the spinal cord, and with scarcely a failure; indeed, I may say without any failure, except in two cases where deep sinuses had formed, which could not be reached by the apparatus. A thin silver plate, no thicker than a sheet of paper, is cut to the exact size and shape of the bed-sore; a zinc plate of about the same size is connected with the silver by fine silver or copper wire, six or eight inches in length. The silver wire is placed in immediate contact with the bed-sore, and the zinc plate on some part of the skin above, a piece of chamois skin soaked in vinegar intervening. This must be kept moist, or there is little or no action of the battery. Within a few hours the effect is perceptible, and in a day or two the cure is complete, in a majority of cases. In a few instances a longer time is required. I have frequently seen bed-sores three or four inches in diameter, and a half inch deep, heal over in forty-eight hours. Mr. Spencer Wells states that he has often witnessed large ulcers covered with granulations within twenty-four hours, and completely filled up and cicatrization begun in forty-eight hours. During his recent visit to this country, I informed him of my experience, and he reiterated his opinion that it was the best of all methods for treating ulcers of indolent character and bed-sores.—*Pacific Med. and Surg. Journal.*

Dr. R. Angus Smith says that the air of houses should not contain carbonic acid enough to give a precipitate when a ten-ounce bottleful is shaken with half an ounce of clear lime-water.—*Med. Times.*

New Method of Treating Small-Pox and Typhoid Fever.—Dr. J. Heine has a long article on this subject in *Virchow's Archiv.* vol. 54. It is in the form of a letter to Virchow, and the treatment described in the paper is quite such as to warrant the author in designating his letter a "schismatic epistle." The treatment recommended is by the external application of solutions of corrosive sublimate, and he claims to have obtained great success by its means in small-pox, especially in petechial or hæmorrhagic small-pox, but also in confluent and other forms. He uses two solutions, one containing 25 and the other 50 grains of corrosive sublimate, in 18 ounces of water and 1 ounce of alcohol. The stronger solution he only uses in petechial small-pox, and continues its use only for a short period ; the weaker is that generally employed. Pieces of clean-washed linen cloth are soaked in the solution, and laid in a double layer on various parts of the body alternately, the parts chiefly chosen being the upper or lower arm, the leg, breast, abdomen, neck ; over the cloth waxed paper is laid in order to prevent evaporation. The average quantity of the weaker solution used in one case, and spread over several weeks, represented an amount of corrosive sublimate equal to 170 to 230 grm. ($5\frac{1}{2}$ to $7\frac{1}{2}$ ounces.)

The author then gives his ideas as to the action of corrosive sublimate on the human body, and he claims for it four properties. 1. Its effect is unsurpassable in all wounds which have taken on a diphtheritic character. 2. It has a similarly powerful effect in causing absorption of all kinds of exudations in every part of the body. 3. It has an undeniable antipyæmic property which influences this disease up to a certain stage. 4. It has the power within 4 to 6 days of arresting or moderating fever symptoms, which depend on general infection of the blood or on local deposits. As showing the last of these properties, the author treats more in detail of the use of the sublimate in typhus abdominalis (typhoid fever), and he asserts that it is able in the initial or more advanced stages of the disease to cut short the fever in the course of 5 to 7 days. The records of four cases with the temperature curves are given, and the amount of corrosive sublimate used in these cases was in grains, 120, 100, 100, and 80, respectively. The solution used was 30 grains to the proportion of water and alcohol given above. The sublimate is sometimes followed by an alarming increase in the diarrhœa, but without tenesmus or hæmorrhage. Its beneficial influence, however, is soon evidenced by the change of the color of the stools from the usual pea-soup character to a saturated bilious color. The other effects of the sublimate as respects urine, nervous symptoms, etc., are also given. The author also claims for the corrosive sublimate a special influence on the action of the heart. The paper concludes with certain considerations as to the present position of medical science. —*Medical Reporter.*

Bullock's blood is now the fashionable remedy among the Parisians for anæmia and phthisis. It is said to be a curious sight to see the number of patients, of both sexes and all ranks and ages, who flock to the slaughter-house every morning to drink of the still foaming blood of oxen slaughtered for the table. The young ladies take it with great facility, and many say that they prefer it to cod liver oil. For the more fastidious, however, a pharmacein has prepared an extract of blood which is administered in the form of pills. Three grains of this extract is said to represent about half an ounce of pure blood. Many cases of anæmia have been cured by this blood treatment, and some phthisical patients greatly benefited, at least as much as they would have been under cod liver oil.

Medical Rank in the Navy.—The *Naval Register*, recently published, gives the following numbers and rank of the Medical Staff: 15 Medical Directors, with the relative rank of Captain; 15 Medical Directors, with the relative rank of Commander; 50 Surgeons, with the relative rank of Lieutenant-Commander; 25 Passed Assistant Surgeons, with the relative rank of Lieutenant; and 52 Assistant Surgeons, with the relative rank of Master.

Among the "volunteer officers" are 2 Acting Passed Assistant Surgeons, and 11 Acting Assistant Surgeons.—*Med. Times.*

Dr. Brown-Sequard having resigned the chair of Comparative and Experimental Physiology in the Parisian Faculty of Medicine, Dr. Vulpian has been appointed in his place. This makes a vacancy in the chair of Pathological Anatomy, which will probably be filled by the nomination of Dr. Charcot, the well-known physician of La Saltpetriere.—*Med. Times.*

The retirement of Dr. J. M. Foltz, late Chief of Bureau of Medicine and Surgery, U. S. Navy, has placed Dr. J. C. Palmer in that position.—*Med. Times.*

Harmless hair-dye is made in Greece from green walnut burs (Juglans regia), by extracting with water and evaporating until the Regianic acid is precipitated as a black powder.

Guarana in Sick Headache.—It is the congestive forms of headache that guarana most frequently succeeds; thus it will sometimes relieve the throbbing headache of the man who "got exceedingly drunk yesterday," and whose "brain is still sticking to the ceiling," with startling rapidity. In such cases the action of the medicine may be seen, for the large injected conjunctival vessels become small by degrees and beautifully less as the drug is absorbed and the cephalalgia departs. This seems to suggest its action as being stimulant to the sympathetic system. If further trial prove this to be so, we must regard it as the opposite in action to the nitrite of amyl.—*Bradley—Brit. Med. Journal, June* 29, '72.

Recurring Tonsillitis.—Dr. Lewin, of Berlin, recommends the application of crystallized chromic acid to those cases of tonsillitis in which a chronic congestion exists, constantly liable on the least provocation, to blaze up into an acute attack. The acid causes ulceration of the tissue to which it is applied, and induration of the surrounding parts to such an extent as to compress the enlarged capillaries beyond the possibility of their subsequently taking on inflammatory hyperæmic action.

In acute painful conditions of these organs, he injects a morphine solution into their substance, and by so doing, greatly lessens the pain and difficulty of deglutition.—*N. W. Med. and Surg. Jour.*

Hare-Lip.—The recommendation has recently been revived, that, after the pins or sutures have been removed from a hare-lip which has been operated upon, collodion should be used to draw and keep the parts closely in apposition. Gauze, or a thin layer of cotton wadding, is saturated with collodion, and while the cheeks are pressed together, this is spread over the lip like a mustache. The contractile power of the collodion is sufficient to hold the newly healed parts together with a considerable degree of firmness; and the advantage is thus obtained, of being able to remove the sutures early, by the third or fourth day.—*N. W. Med. and Surg. Jour.*

The Augusta Medical College of Georgia has been made the Medical Department of the University of Georgia; a compliment well bestowed and fully merited.

In the third week of its present regular session, there were 205 students in the Louisville Medical College, in that city.

